# Sleep duration, daytime napping, markers of obstructive sleep apnea and stroke in a population of southern China

**DOI:** 10.1038/srep34689

**Published:** 2016-10-04

**Authors:** Ye Wen, Fu-Hua Pi, Pi Guo, Wen-Ya Dong, Yu-Qing Xie, Xiang-Yu Wang, Fang-Fang Xia, Shao-Jie Pang, Yan-Chun Wu, Yuan-Yuan Wang, Qing-Ying Zhang

**Affiliations:** 1 Department of Preventive Medicine, Shantou University Medical College, Shantou, Guangdong 515041, China; 2 Department of Sports, Shantou University Medical College, Shantou, Guangdong 515041, China; 3 Department of neurology, The First Affiliated Hospital of Shantou University Medical College, Shantou, Guangdong 515041, China

## Abstract

Sleep habits are associated with stroke in western populations, but this relation has been rarely investigated in China. Moreover, the differences among stroke subtypes remain unclear. This study aimed to explore the associations of total stroke, including ischemic and hemorrhagic type, with sleep habits of a population in southern China. We performed a case-control study in patients admitted to the hospital with first stroke and community control subjects. A total of 333 patients (*n* = 223, 67.0%, with ischemic stroke; *n* = 110, 23.0%, with hemorrhagic stroke) and 547 controls were enrolled in the study. Participants completed a structured questionnaire to identify sleep habits and other stroke risk factors. Least absolute shrinkage and selection operator (Lasso) and multiple logistic regression were performed to identify risk factors of disease. Incidence of stroke, and its subtypes, was significantly associated with snorting/gasping, snoring, sleep duration, and daytime napping. Snorting/gasping was identified as an important risk factor in the Lasso logistic regression model (Lasso’ *β* = 0.84), and the result was proven to be robust. This study showed the association between stroke and sleep habits in the southern Chinese population and might help in better detecting important sleep-related factors for stroke risk.

As a major public health concern, stroke is the second leading cause of death and the third most important cause of disability-adjusted life-years worldwide^1^. Due to the negative impact of rapid urbanization, aging of the population and the relatively early age at which stroke occurs, China and India, the world’s two most populous countries, however, are likely to bear the major burden^1^,^2^.

Sleep is a natural and essential physiological process crucial for homeostasis. Various studies have examined the association between sleep duration and risk of stroke, but the results are inconsistent and dependent on social, cultural, and demographic factors^3^,^4^,^5^. A recent meta- analysis of 15 prospective cohorts showed that both short and long durations of sleep were associated with high risk of stroke, presenting a U-shaped relationship^6^. Other studies reported strong effects for long sleep only, indicating a J-shaped relationship^3^,^5^. In addition, studies investigating ischemic and hemorrhagic stroke separately are rare and evidence from Chinese populations is limited.

Daytime napping confers health benefits through a postulated “stress-releasing” mechanism^7^. Meanwhile, characterized by daytime napping, daytime sleepiness has been treated as an indicator of poor sleep quality or potential health disorders^8^,^9^. Given the high prevalence of daytime napping in elder Chinese, it is important to have a further understanding in the relationship between daytime napping and stroke risk in elder population.

Significant association between snoring and a range of health problems had been described in many studies^10^,^11^,^12^. As a major type of sleep-disordered breathing, obstructive sleep apnea (OSA) was a condition characterized by repetitive episodes of partial/complete airway obstructions and hypopneas during sleep, these phenomena may bring out a series of deleterious effects, such as hypoxemia and hypercapnia. Instead of polysomnography, which is the gold standard for diagnosis of OSA, snoring is often used as indicator of OSA in epidemiological studies as a quick, easy and inexpensive strategy^13^. However, snoring is also common in patents without OSA^14^, consequently, related researches that attended to explore the associations between stroke events and new OSA-related variables: snorting/gasping and breathing stops/choking/struggling for breath would be needed. Once the relationship between stroke outcomes and self-reported variables has been determined, their significant association can be applied by clinical and research workers in better targeting vulnerable populations.

Over the past 30 years, the lifestyle of people has changed in China^15^. Nevertheless, Shantou, located on the southeast coast of Guangdong Province of China and with more than 5 million population, retain their local dialect and unique but relatively stable life styles, particularly the elderly^16^. In this study, we aimed to identify potential sleep factors associated with stroke events and further, investigate whether those factors were independently associated with the risk of stroke in a population in southern China.

## Materials and Methods

### Study population

The present study included 333 patients (*n* = 223, 67.0%, with ischemic stroke; *n* = 110, 23.0%, with hemorrhagic stroke) and 547 controls from Shantou between May 2011 and December 2013. Patients with first acute stroke admitted to the First Affiliated Hospital of Shantou University Medical College and Chaonan Minsheng Hospital were considered. The patient inclusion criteria were as follows: (1) age of 18 years or older, (2) hospital admission within 72 hours of symptom onset, and (3) availability of CT or MRI neuroimaging information. The exclusion criteria were as follows: (1) inability to communicate, (2) presence of tumor, presently in hospital for acute coronary syndrome, or subdural hemorrhage, and (3) inability to provide consent for inclusion in the study. Controls without a previous history of stroke were recruited from the community. The exclusion criteria for controls were identical to those described for patients with stroke. Stroke is defined as a clinical syndrome caused by sudden rupture or occlusion of blood vessels in the brain, and is characterized by loss of cerebral function and rapid development symptoms, which persist for more than 24 hours and may possibly cause death^17^.

### Procedures

Structured questionnaires and physical examinations were given and performed, respectively, in the stroke patients and controls by trained personnel. We chose all covariates based on literature^18^,^19^. Information about demographic factors, history of hypertension, diabetes mellitus, physical measurements, socioeconomic status, lifestyle factors, psychosocial factors, physical activities, and heart disease (atrial fibrillation, previous myocardial infarction, rheumatic valvular disease, and prosthetic heart valve), history of medications, tobacco, alcohol use, dietary habits and sleep habits were investigated as potentially associated factors. Questions about sleep habits included the following: (a) “on the average, how many hours of sleep do you get at night?”; (b) “do you usually sleep during the day?”; (c) “have you been told that you have the following symptoms (snoring; snorting/gasping; breathing stops/choking/struggling for breath) during sleep?”; if the answer is yes, then the symptoms were quantified as “less than once a week”, “once or twice per week”, “3–4 times per week”, or “5–7 times per week”.

This study was conducted in accordance with the Declaration of Helsinki. Informed consent was obtained from the participants and the study was approved by the ethics committees of the First Affiliated Hospital of Shantou University Medical College and Chaonan Minsheng Hospital.

### Statistical analysis

Descriptive statistics were used to evaluate the characteristics of the subjects. Data of continuous variables were presented as mean (SD), and differences were assessed by *t* tests. Categorical variables were expressed as number (percentage) and tested using Pearson’s *χ*^2^ analysis.

The general logistic regression model was used to calculate the odds ratios (ORs) and 95% confidence intervals (95% CIs). The association between sleep habits and stroke was assessed with different multivariable logistic regression. Covariates in model A included age, sex, sleep duration, daytime napping, snoring, and snorting/gasping; model B further adjusted for education, smoking, alcohol, vegetables, fruits consumption status, and history of diabetes, based on the fact that those covariates are associated with obesity and hypertension, which may act as partial mediators of the relationship between sleep habits and stroke risk, we further adjusted for the effects of history of hypertension and body-mass index (BMI) in model C. Finally, physical activity, sleep quality, and psychosocial factors were further adjusted in model D. Each model adjusted for the other covariates, except for itself. We also tested the multiplicative interactions between sleep habits and age, gender, history of hypertension, and BMI on the risk of stroke and found that gender acted as an effect modifier between snoring and stroke risk (P < 0.05). We then stratified the study population by gender and performed the full adjusted logistic regression analyses with each.

SPSS software 20.0 (SPSS, Inc., Chicago, IL, USA) was used for statistical analyses. All statistical tests were two-sided, and a *P*-value <0.05 was considered statistically significant.

Penalized likelihood-based methods have drawn much attention recently^20^. Since the variables are generally numerous, a suitable variable selection operator should be proposed to search the most important risk factors contributing to stroke events and avoid using a complex model. Thus, the least absolute shrinkage and selection operator (Lasso), which has been widely used in variable selection^21^,^22^, was employed to select the most important risk factors for stroke in our study.

Suppose that we have an input n × p matrix 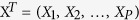, and aim to predict a n × 1 binary response vector Y, the logistic regression is defined as:


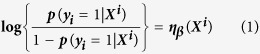


where 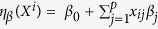, *β*_0_ is the intercept and *β*_*j*_ is the parameter corresponding to *x*_*j*_.

The Lasso estimator is defined as:


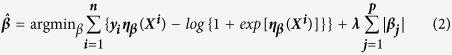


where λ is the tuning parameter. Making λ sufficiently large will cause some of the coefficients to be exactly zero. Thus the Lasso performs a continuous subset selection.

Parameter estimations in the Lasso were computed via a cyclical coordinate descent algorithm^23^. A 10-fold cross-validation was performed to estimate the error rate for each model. In order to confirm the robustness of the Lasso method, a simulation study was conducted. First, we specified various sample sizes (*N* = 100, 250 and 500), before we randomly resampled 100 sets of bootstrapped and permutated data with various sample sizes from the survey data. Finally, after conducting the Lasso logistic regression model, the significant covariates and the corresponding frequency outputs of covariates were recorded. The Lasso logistic regression model was established using the glmnet package within R version 3.1.3.

## Results

### General information

Overall, the present study included 880 adults (56.36% male), with a mean age of 64.32 (SD: 12.41) years. Among the 333 patients, 223 (67.0%) had ischemic stroke and 110 (23.0%) had intracerebral hemorrhagic stroke. Additional ischemic stroke etiologies included cardioembolism (*n* = 6, 1.8%), large vessel (*n* = 68, 20.4%), and small vessel (*n* = 133, 9.9%) (see Supplementary Table S1). The number of subjects reported to have a history of atrial fibrillation or flutter, rheumatic valvular heart disease and prosthetic heart valve in our study was 2 (<1%), 1 (<1%), and 1 (<1%), respectively, and few of the participants had a history of lipid-lowering medications (*n* = 6, <1%). Additionally, 146 (16.6%) of the participants reported using of antihypertensive medications, with no significant difference between cases and controls (*n* = 60, 18.0% vs *n* = 86, 15.7%, P = 0.401).

The baseline characteristics of the participants are summarized in Table 1. There were approximately equal percentage of men and women between cases and controls. Cases were older than controls and more likely to have a history of hypertension but less likely to have a history of migraine and less educated. Besides, cases were more likely to be current alcohol consumers, smokers, and physically inactive. Cases and controls did not differ in WHR (waist hip rate), history of diabetes, income level, marital status, and depressive symptoms (all P > 0.05).

Half of the participants reported taking daytime naps, and 15.9%, 77.5%, and 6.6% of the participants reported sleeping <7 h, 7 to <9 h, and >9 h per day, respectively. Among the participants, 47.5% reported that they do not snore, 24.1% reported snoring sometimes, and 28.4% reported about frequent snoring. The percentage of participants who do not snort/gasp was 69.3% for the total study population.

### Identification of stroke related factors

**Figure 1:**
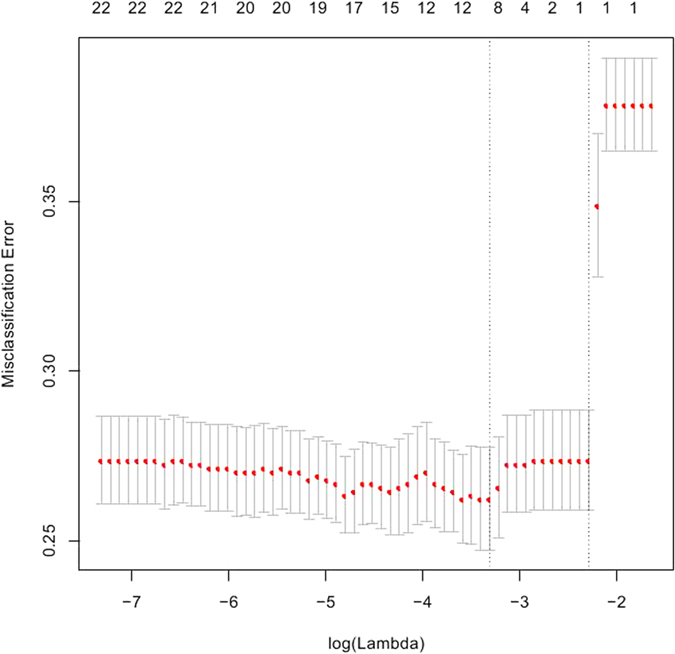
Ten-fold cross-validation misclassification error rates with error bars of the Lasso logistic regression model across different values of the tuning parameter λ (log-scale). The total number of covariates is 23. The minimum cross-validated error reached when the log of λ is −3.31. When the log of λ = −2.29, the error is within 1 standard error of the minimum cross-validated error.

**Figure 2:**
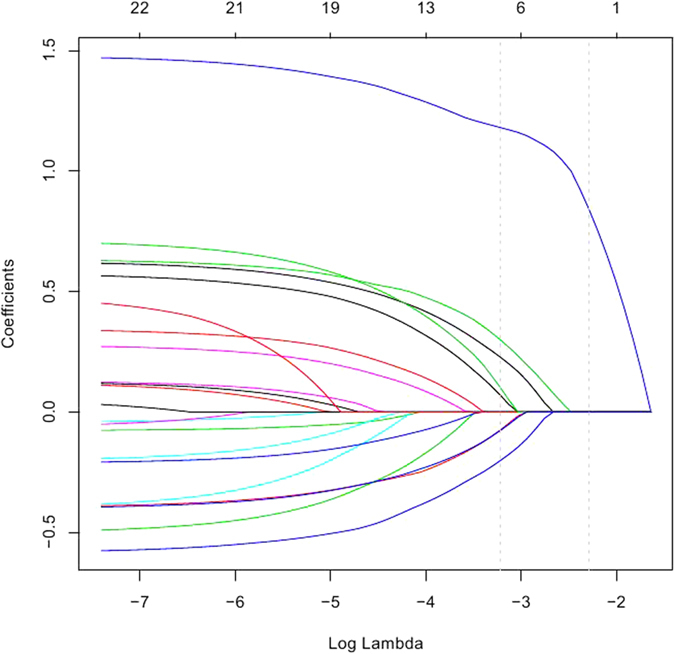
The path of the parameter estimated over a grid of values for λ. The numbers of selected covariates were eight and one for the two tuning parameter λ.

Figure 1 showed the cross-validation error rates and the numbers of selected covariates at a grid values of λ for the Lasso models. According to the results of 10-fold cross-validation, the most suitable tuning parameter λ that given minimum cross-validated error was −3.31(log scale), the number of selected covariates was eight and the coefficients of covariates was showed in Table 2. When the tuning parameter reached its largest scale (with a cross-validated error within 1 standard error of the minimum), snorting/gasping was the only selected covariate (Lasso’s *β* = 0.84). The corresponding regularization path was presented in Fig. 2.

**Figure 3:**
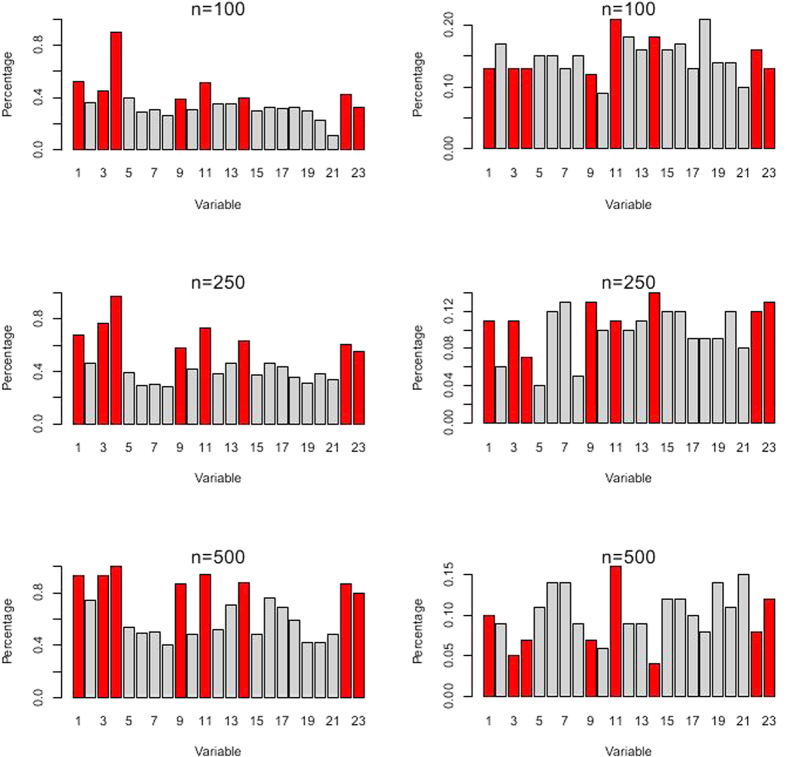
The Lasso method with λ = −3.31 (log-scale) on the simulated data. In each scenario, the percentage of the bootstrapped data is on the left, whereas the percentage of the permuted data is on the right. The red bar represents the covariate of daytime napping, snoring, snorting/gasping, BMI, hypertension, consumption of vegetables, consumption of fruits, and education status in turn.

Figure 3 presented the validation results of the Lasso model with the most suitable tuning parameter. The significant eight covariates were selected almost all the time in the bootstrapped data, while in the permuted data, the random and unpredictable selection of variables demonstrated that the previously association among the response and the covariates disappeared. Hence, the results of the Lasso method were deemed to be robust.

**Figure 4:**
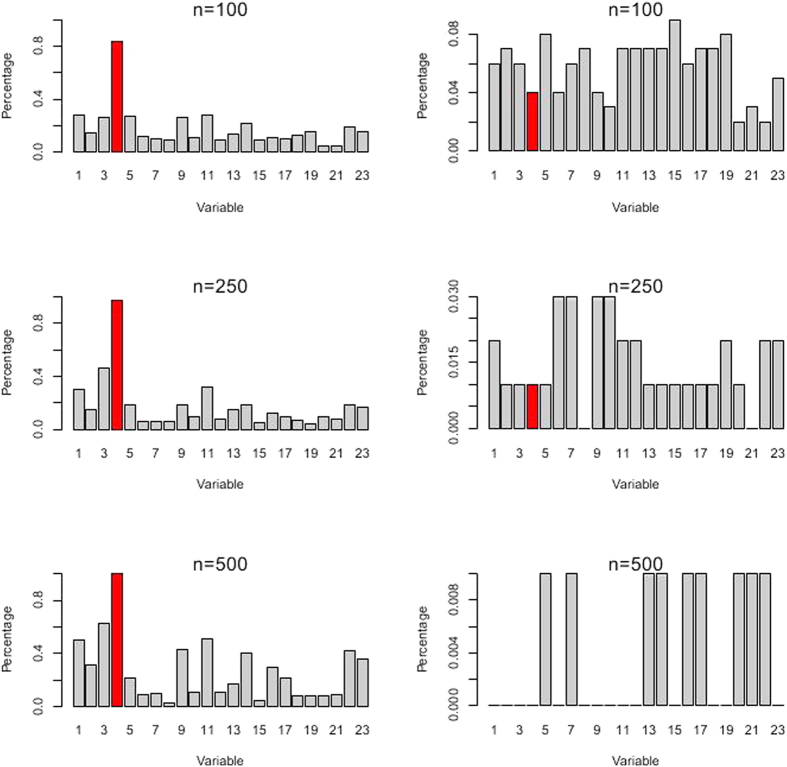
The Lasso method with λ = −2.29 (log-scale) on the simulated data. In each scenario, the percentage of the bootstrapped data is on the left, whereas the percentage of the permuted data is on the right. The red bar represents the covariate of snorting/gasping.

We noted that, when the tuning parameter reached its largest scale, snorting/gasping was the only selected covariate, meanwhile, snorting/gasping was a strong risk factor in the logistic model (Table 2). Additionally, the same signs of the coefficients reflected that these two models found the similar effects modified by snorting/gasping. The validation result of the Lasso model with the largest value of λ was also robust as shown in Fig. 4.

### Sleep habits factors and stroke risk

Multiple multivariable adjusted ORs for sleep duration and stroke were presented in Table 3. Compared with sleep duration of 7 to <9 h per night, long sleep duration (≥9 h) was independently associated with increased risk of stroke (Table 3). The adjusted OR was 2.53 (95% CI: 1.51 to 4.25) for long sleep duration in model A. The relationship attenuated in model B, and further diminished in model C. The result remained significant after adjustment for all covariates (OR: 2.00, 95% CI: 1.16 to 3.46) but was marginally stronger in hemorrhagic stroke (OR: 2.25, 95% CI: 0.99 to 5.10) than ischemic stroke (OR: 1.94, 95% CI: 1.07 to 3.53).

As shown in Table 3, lack of daytime napping was independently associated with elevated risk of stroke. In comparison with the result in Model A, the above associations slightly attenuated in model B and C. After full adjustment for potential confounders, participants without daytime napping had a higher risk of stroke than nappers, displaying an OR of 1.73 (95% CI: 1.24 to 2.43). The results differed in stroke subtypes, with a higher risk for hemorrhagic stroke (OR: 2.99, 95% CI: 1.73 to 5.16) than for ischemic stroke (OR: 1.46, 95% CI: 1.00 to 2.12). Similarly, individuals reported snorting/gasping were also at increased risk of stroke with a full adjustment OR of 4.18 (95% CI: 2.62 to 6.66) and the relationship was more prominent in hemorrhagic stroke (OR: 5.79, 95% CI: 2.75 to 12.16) than in ischemic stroke (OR: 3.73, 95% CI: 2.22 to 6.26).

After adjustment for all the potential confounders, both frequent snorers and occasional snorers were independently associated with total stroke risk and ischemic stroke (Table 4). The above associations were significant among men, displaying ORs of 2.07 (95% CI: 1.09 to 3.96) and 2.27 (95% CI: 1.14 to 4.52) for frequent snorers and occasional snorers respectively. In addition, significant associations were observed between occasional snoring (OR: 3.68, 95% CI: 1.24 to 10.91) and total stroke among women.

Subgroup analyses for sleep habits with respect to etiological stroke subtypes (large vessel and small vessel) were shown in Supplementary Table S2. In addition, sensitivity analysis was performed by sequentially excluded participants with a history of heart disease, antihypertensive medications and lipid-lowering medications at each turn and the results did not alter materially (see Supplementary Table S3).

## Discussion

This study provided essential information on the association between stroke and risk factors especially concerning the sleep habits of the population in southern China. The traditional univariate analysis detected significant association with fourteen risk factors while the Lasso logistic regression produced a sparser model with eight risk factors. Multiple multivariable adjusted logistic regressions analyses supported the roles of sleep habits, including sleep duration, daytime napping, snoring, snorting/gasping as significant risk factors for all stoke, ischemic stroke and hemorrhagic stroke. For the Lasso method, the results emphasized the role of snorting/gasping in elevating stroke risk. This study might help in better detecting the important risk factors and also indicated that adjusting sleep habits especially reducing the frequency of snorting/gasping may be considered as an effective intervention strategy to weaken the risk of stroke.

We observed a J-shaped relationship between sleep duration and total stroke risk, after controlling for potential confounders in the southern Chinese population. The association was consistently observed in both ischemic and hemorrhagic stroke. Our findings are compatible with previous investigations in British adults^3^, US^24^, and Singaporean-Chinese populations^4^ which showed an HR of 1.46 (95% CI: 1.08 to 1.98), 2.22 (95% CI: 1.69 to 2.91), and 1.54 (95% CI: 1.28 to 1.85) for long sleep duration, respectively. This result is also consistent with the JACC study^25^, where long duration of sleep was associated with total stroke and ischemic stroke in a large cohort of Japanese men and women, our study extended the association to hemorrhagic stroke also.

The mechanisms for the association between long sleep duration and increased risk of stroke have not been fully elucidated. It is possible that the selection of taking longer nighttime sleep duration among elder people was explained by fatigue or driven by disease burden and co-morbidities^6^, although we have included common chronic diseases in our analysis, other diseases were possibly responsible for the prolonged sleep duration. Therefore longer sleep duration could be a subtle marker of the disease, rather than as a cause of stroke. For short sleep duration, the lack of association with stroke risk is possibly due to the modified effects of factors including daytime naps, sleep disorders, and sleep quality. However, generalizing the null results of our study in an middle-aged and older Chinese population to other populations is cautioned, since subjects with middle-age and older have been shown to have more opportunities in taking daytime napping^26^.

A beneficial effect of daytime napping for stroke and its subtypes was shown in our research. Daytime napping is a well-accepted routine among elder Chinese, as it may relieve stress and depression^27^. Although preceding prospective studies demonstrated adverse health outcomes of daytime napping^28^, our result is consistent with physiological data from short-term laboratory studies concerning the beneficial effects of daytime napping on psychomotor vigilance task, daytime sleepiness, and neurobehavioral performance^29^,^30^. However, we did not distinguish between short and long daytime nap duration, which remains to be elucidated in further studies.

Snoring, a major marker of OSA, also affects about 40% of men and 20% of women without OSA, and is demonstrated to be closely associated with risk of stroke^10^,^31^. The same view can be also put forward in the present study. In addition, a novel results of this study was the robust association between snorting/gasping and stroke risk. Self-reported snoring and snorting/gasping each provide an aspect of sleep apnea. Animal experiments^32^ among rabbits demonstrated that the resistance to airflow in the upper airways and subsequent production of energy from the vibrations of the pharyngeal wall may be transmitted to the proximal carotid artery wall and lumen. Such vibrations may trigger a cascade effect that can lead to arterial endothelial cell dysfunction and subsequent atheroma formation closely associated with stroke^33^. In addition, experimental evidence has linked the deleterious effect of partial/complete airway obstructions, and accompanying hypoxemia and hypercapnia to the cardiovascular system through several processes, including activation of the sympathetic nervous system, endothelial dysfunction, inflammation, oxidative stress, vascular smooth muscle cell activation, platelet activation and thrombosis^14^.

The Lasso logistic model has been used in variable selection in many fields as it can effectively exclude some irrelevant variables and produce sparse estimations and good asymptotic properties^34^, but this study was the first to apply Lasso in the identification of the most important risk factors that contribute to elevated stroke risk in a case-control design. Since a structured questionnaire always include a large number of variables which can lead to the problem of multicollinearity, both the consistency of the Lasso method and the traditional Logistic regression as well as the stability verified by simulation experiments in our research showed that it was appropriate for solving that issue and may enhance our ability for mining new information on large epidemiological investigation.

Several limitations of this study should be considered. First, a potential disadvantage was the relatively small sample size in the present study, an epidemiological investigation with larger sample size is needed to verify the results in the future. Second, the population our study based on might not be representative of the general southern Chinese population. Third, although our study have taken a large number of potential confounders into consideration, we could not completely rule out the possibility of unmeasured confounding. Also, sleep habits were self-reported and were difficult to interpret and quantify, as a result of participant’ perception or night-to-night sleep variation. However, residents in Shantou region have relative steady rhythm of life, and the interviewers were trained to conduct thorough interviews on sleep habits, besides, the study included comprehensive information on both sleep duration and the quality of sleep that can possibly lead to more accurate results. Finally, while the case-control design had the superiority of being cost effective, resource and time efficient, potentially subject bias could have been introduced by patient recall, and causal associations cannot be inferred given the nature of the cross sectional design.

In summary, the results of this study appear to support the role of sleep habits as a fundamental determinant of the elevated risk of stroke in southern China. Snorting/gasping, snoring frequency, lack of daytime napping, and long sleep duration are statistically significant independent risk factors for stroke and its subtypes.

## Additional Information

**How to cite this article**: Wen, Y. *et al*. Sleep duration, daytime napping, markers of obstructive sleep apnea and stroke in a population of southern China. *Sci. Rep*. **6**, 34689; doi: 10.1038/srep34689 (2016).

## Supplementary Material

Supplementary Information

## Figures and Tables

**Table 1:** Baseline characteristics of participants.

Characteristics	Cases n (%) (*N* = 333)	Controls n (%) (*N* = 547)	*χ*^2^/*t*	*P*-value
Male	181 (54.4)	315 (57.6)	0.88	0.348
Age (mean ± SD), year	64.97 (13.17)	63.93 (11.92)	5.85	**0.016**
BMI			12.37	**0.002**
<18.5	20 (6.0)	22 (4.0)		
18.5–25	244 (73.3)	354 (64.7)		
≥25	69 (20.7)	171 (31.3)		
WHR			3.18	0.204
<0.68	142 (42.6)	204 (37.3)		
0.68–0.90	93 (27.9)	154 (28.2)		
≥0.90	98 (29.4)	189 (34.6)		
Hypertension	154 (46.2)	186 (34.0)	13.09	**<0.001**
Diabetes	40 (12.0)	63 (11.5)	0.05	0.825
Migraine	57 (17.1)	134 (24.5)	6.63	**0.010**
Smoking			9.13	**0.010**
Formerly	13 (3.9)	48 (8.8)		
Currently	146 (43.8)	206 (37.7)		
Never	174 (52.3)	293 (53.6)		
Alcohol			9.22	**0.010**
Formerly	15 (4.5)	36 (6.6)		
Currently	63 (18.9)	65 (11.9)		
Never	255 (76.6)	446 (81.5)		
Physical activity			18.89	**<0.001**
Mainly sedentary	151 (45.3)	246 (45.0)		
Mild	164 (49.2)	222 (40.6)		
Moderate/strenuous	18 (5.4)	79 (14.4)		
Consumption of vegetables	30 (9.0)	83 (15.2)	7.09	**0.008**
Consumption of fruits	143 (42.9)	164 (30.0)	15.31	**<0.001**
Education status			30.09	**<0.001**
Illiteracy	99 (29.7)	125 (22.9)		
Elementary education	224 (67.3)	345 (63.1)		
Trade school/college/university	10 (3.0)	77 (14.1)		
Income			0.33	0.849
Higher	156 (46.8)	264 (48.3)		
Middle	122 (36.6)	190 (34.7)		
Lower	55 (16.5)	93 (17.0)		
Single or divorced	26 (7.8)	59 (10.8)	2.10	0.147
General stress	3 (0.9)	6 (1.1)	0.08	0.779
Depression	6 (1.8)	11 (2.0)	0.05	0.827

*N*, number; *P*-value, significance of the difference between group means and frequency determined by t-test and chi-square test.

**Table 2:** Comparison of the estimates of sleep-related factors from the logistic regression model and the Lasso logistic regression model.

Variable name	Case n (%)	Control n (%)	Lasso’s *β*	Logistc’s *β*	*P*-value
Sleep duration					
7–<9	230 (69.1)	424 (77.5)			
<7	56 (16.8)	87 (15.9)		−0.66	0.781
≥9	47 (14.1)	36 (6.6)		0.69	**0.013**
Daytime napping			0.25		
Yes	132 (39.6)	308 (56.3)			
No	201 (60.4)	239 (43.7)		0.55	**0.001**
Snoring			0.32		
Never	92 (27.6)	326 (59.6)			
Occasional	192 (57.7)	150 (27.4)		0.54	**0.027**
Frequent	49 (14.7)	71 (13.0)		0.74	**0.007**
Snorting/gasping			1.19		
No	152 (45.6)	458 (83.7)			
Yes	181 (54.4)	89 (16.3)		1.43	**<0.001**

**Table 3:** Associations between sleep habits (sleep duration, daytime napping, and snorting/gasping) and stroke by subgroups, with different levels of adjustments.

Sleep habits	Sleep duration			Daytime napping		Snorting/gasping	
	7–<9 h	<7 h	≥9 h	Yes	No	No	Yes
Total stroke							
Model A	1.00	0.94 (0.62**–**1.44)	**2.53 (1.51–4.25)**	1.00	**2.00 (1.46–2.72)**	1.00	**4.03 (2.65–6.13)**
Model B	1.00	0.89 (0.57**–**1.38)	**2.29 (1.34–3.92)**	1.00	**1.75 (1.26–2.43)**	1.00	**4.50 (2.87–7.05)**
Model C	1.00	0.92 (0.59**–**1.44)	**2.15 (1.24–3.70)**	1.00	**1.74 (1.25–2.42)**	1.00	**4.21 (2.66–6.67)**
Model D	1.00	0.94 (0.59**–**1.49)	**2.00 (1.16–3.46)**	1.00	**1.73 (1.24–2.43)**	1.00	**4.18 (2.62–6.66)**
Ischemic stroke							
Model A	1.00	0.89 (0.55**–**1.44)	**2.54 (1.43–4.51)**	1.00	**1.69 (1.19–2.39)**	1.00	**3.93 (2.46–6.27)**
Model B	1.00	0.85 (0.51**–**1.39)	**2.16 (1.20–3.88)**	1.00	**1.49 (1.03–2.14)**	1.00	**4.16 (2.53–6.86)**
Model C	1.00	0.87 (0.52**–**1.45)	**2.05 (1.13–3.70)**	1.00	**1.47 (1.01–2.13)**	1.00	**3.80 (2.28–6.32)**
Model D	1.00	0.87 (0.51**–**1.46)	**1.94 (1.07–3.53)**	1.00	1.46 (1.00**–**2.12)	1.00	**3.73 (2.22–6.26)**
Hemorrhagic stroke							
Model A	1.00	0.95 (0.52**–**1.76)	**2.52 (1.20–5.29)**	1.00	**3.00 (1.86–4.84)**	1.00	**4.23 (2.27–7.88)**
Model B	1.00	0.81 (0.42**–**1.55)	**2.58 (1.16–5.75)**	1.00	**2.86 (1.70–4.79)**	1.00	**5.68 (2.84–11.37)**
Model C	1.00	0.82 (0.42**–**1.62)	**2.43 (1.09–5.44)**	1.00	**2.86 (1.68–4.87)**	1.00	**5.44 (2.65–11.15)**
Model D	1.00	0.97 (0.47**–**1.97)	2.25 (0.99**–**5.10)	1.00	**2.99 (1.73–5.16)**	1.00	**5.79 (2.75–12.16)**

Model A adjusted for age, sex, sleep duration, daytime napping, snoring, and snorting/gasping.

Model B adjusted for the variables in model A plus education, smoking, alcohol, vegetables, fruits consumption status, and history of diabetes.

Model C adjusted for the variables in model B plus history of hypertension and body-mass index.

Model D adjusted for the variables in model C plus physical activity, sleep quality, and psychosocial factors.

Each model adjusted for the other covariates, except for itself.

**Table 4:** Associations between snoring and stroke by subgroups.

Sleep habits	Snoring		
	Never	Occasional	Frequent
Total stroke*	1.00	**1.72 (1.06–2.79)**	**2.09 (1.22–3.58)**
Ischemic stroke*	1.00	**1.91 (1.11–3.29)**	**1.87 (1.02–3.45)**
Hemorrhagic stroke*	1.00	1.27 (0.58**–**2.76)	2.24 (0.97**–**5.20)
Male#	1.00	**2.07 (1.09–3.96)**	**2.27 (1.14–4.52)**
Female#	1.00	1.69 (0.72**–**4.02)	**3.68 (1.24–10.91)**

^*^Adjusted for covariates in model D.

^#^Adjusted for covariates in model D except for age.
