# Supplementary material for: Sleep duration, daytime napping, markers of obstructive sleep apnea and stroke in a population of southern China
Source: Sci Rep. 2016 Oct 4;6:34689. doi: 10.1038/srep34689 (PMC5048149; doi:10.1038/srep34689)
Supplement: Supplementary Information [file srep34689-s1.doc]

Sleep duration, daytime napping, markers of obstructive sleep apnea and stroke in a population of southern China

Ye Wen ^a^, MD, Fu-Hua Pi ^b^, BA, Pi Guo ^a^, PhD, Wen-Ya Dong ^a^, Yu-Qing Xie ^a^, Xiang-Yu Wang ^a^, Fang-Fang Xia ^a^, Shao-Jie Pang ^a^, Yan-Chun Wu ^a^, Yuan-Yuan Wang ^a^, Qing-Ying Zhang ^a,*^, PhD

^a^ *Department of Preventive Medicine, Shantou University Medical College, Shantou, Guangdong 515041, China*

^b^ *Department of Sports, Shantou University Medical College Shantou, Guangdong 515041, China*

^c^ *Department of neurology, The First Affiliated Hospital of Shantou University Medical College Shantou, Guangdong 515041, China*

**^*^Correspondence to:** Department of Preventive Medicine (Q –Y Z), Shantou University Medical College, Shantou, Guangdong 515041, China. Fax number: 86-754-88566774 E-mail: qyzhang@stu.edu.cn (Q –Y Z).

Subtitle: sleep characteristics and stroke risk.

Itemized list of the tables

Supporting Information in File S1: Contains Tables S1 to S3

Supplementary Table S1. Clinical characteristics of cases.

| TOAST^*^ classification | n | (%) |
| --- | --- | --- |
| Cardioembolism | 6 | 1.8 |
| Large vessel | 68 | 20.4 |
| Small vessel | 133 | 39.9 |
| Other | 4 | 1.2 |
| Undetermined | 122 | 36.6 |
| ^*^TOAST: the Trial of ORG 10172 in Acute Stroke Therapy. | | |

Supplementary Table S2: Associations between sleep habits and stroke by etiological stroke subtypes.

|  | Large vessel  N=68 | Small vessel  N=133 | Others*  N=132 |
| --- | --- | --- | --- |
| Sleep duration |  |  |  |
| 7 – < 9 h | 1.00 | 1.00 | 1.00 |
| < 7 h | 0.95(0.43-2.09) | 0.58(0.29-1.16) | 0.94(0.59-1.49) |
| ≥ 9 h | 2.14(0.85-5.38) | 1.57(0.74-3.34) | **2.00(1.16-3.46)** |
| Daytime napping |  |  |  |
| Yes | 1.00 | 1.00 | 1.00 |
| No | 1.69(0.92-3.09) | **1.86(1.16-3.00)** | **1.73(1.24-2.43)** |
| Snorting/gasping |  |  |  |
| No | 1.00 | 1.00 | 1.00 |
| Yes | **4.24(1.82-9.91)** | **3.34(1.81-6.17)** | **4.18(2.62-6.66)** |
| Snoring |  |  |  |
| Never | 1.00 | 1.00 | 1.00 |
| Occasional | 1.62(0.66-3.97) | **2.50(1.31-4.78)** | **1.72(1.06-2.79)** |
| Frequent | 1.29(0.46-3.62) | **2.23(1.05-4.72)** | **2.09(1.22-3.58)** |
| *Others: including categories of cardioembolism, other and undetermined by the TOAST classification. | | | |

Supplementary Table S3: Risk of stroke associated with sleep habits by source of controls (participants without a history of heart disease, participants without a history of antihypertensive treatment, and participants without a history of hypolipidemic treatment).

| Sleep habits | Participants without heart disease  N= 876 | Participants without a history of hypolipidemic medications  N=874 | Participants without a history of lipid-lowering medications  N=734 |
| --- | --- | --- | --- |
| Sleep duration |  |  |  |
| 7 – < 9 h | 1.00 | 1.00 | 1.00 |
| < 7 h | 0.94(0.59-1.50) | 0.95(0.60-1.51) | 1.13(0.67-1.91) |
| ≥ 9 h | **2.00(1.16-3.47)** | **1.99(1.15-3.45)** | **1.91(1.05-3.47)** |
| Daytime napping |  |  |  |
| Yes | 1.00 | 1.00 | 1.00 |
| No | **1.71(1.22-2.40)** | **1.73(1.23-2.42)** | **1.73(1.18-2.54)** |
| Snorting/gasping |  |  |  |
| No | 1.00 | 1.00 | 1.00 |
| Yes | **4.14(2.60-6.60)** | **4.16(2.61-6.63)** | **4.6(2.68-7.88)** |
| Snoring |  |  |  |
| Never | 1.00 | 1.00 | 1.00 |
| Occasional | **1.71(1.06-2.77)** | **1.70(1.05-2.76)** | 1.68(0.97-2.93) |
| Frequent | **2.08(1.22-3.56)** | **2.08(1.21-3.56)** | **2.09(1.13-3.88)** |
| *Other: including categories of cardioembolism, other and undetermined by the TOAST classification. | | | |
